# Weight Loss Is a Strong Predictor of Memory Disorder Independent of Genetic Influences

**DOI:** 10.3390/genes14081563

**Published:** 2023-07-31

**Authors:** Sunny Chen, Sara M. Sarasua, Nicole J. Davis, Jane M. DeLuca, Stephen M. Thielke, Chang-En Yu

**Affiliations:** 1 Geriatric Research, Education, and Clinical Center, Puget Sound VA Medical Center, Seattle, WA 98108, USA; changeyu@uw.edu; 2 Healthcare Genetics Program, School of Nursing, Clemson University, Clemson, SC 29634, USA; smsaras@clemson.edu (S.M.S.); njd@clemson.edu (N.J.D.); jdeluca@clemson.edu (J.M.D.); 3 Department of Psychiatry and Behavioral Sciences, University of Washington School of Medicine, Seattle, WA 98195, USA; sthielke@uw.edu; 4 Department of Medicine, Division of Gerontology and Geriatric Medicine, University of Washington, Seattle, WA 98195, USA

**Keywords:** *APOE*, body mass index, dementia, memory disorder, weight loss

## Abstract

Background: Past studies identified a link between weight loss and dementia, but lacked consistent conclusions. We sought to establish this link by examining the weight change profiles before and after dementia diagnosis. Methods: Using data from the Health and Retirement Study (1996–2020), we examined 13,123 participants. We conducted a nested case–control analysis to assess differences in biennial weight change profile while controlling for BMI, longevity polygenic risk scores, and *APOE* gene variants. Results: Participants with a memory disorder lost weight (−0.63%) biennially, whereas those without a diagnosis did not (+0.013%, *p*-value < 0.0001). Our case–control study shows a significant difference (*p*-value < 0.01) in pre-dementia % weight changes between the cases (−0.29%) and controls (0.19%), but not in post-dementia weight changes. The weight loss group have the highest risk (OR = 2.01; *p*-value < 0.0001) of developing a memory disorder compared to the stable weight and weight gain groups. The observations hold true after adjusting for BMI, longevity polygenic risk scores, and *APOE* variant in a multivariable model. Conclusions: We observe that weight loss in dementia is a physiological process independent of genetic factors associated with BMI and longevity. Pre-dementia weight loss may be an important prognostic criterion to assess a person’s risk of developing a memory disorder.

## 1. Introduction

Meta-analyses suggest that unintentional weight loss has a significant positive correlation with higher risk of all-cause mortality and morbidity regardless of age, baseline body mass index (BMI) and health status, sex, and race [1,2]. The most common reasons for weight loss in the elderly are linked to nonmalignant gastrointestinal disease, psychiatric conditions, and social factors; however, there are approximately 28% of cases that are associated with unidentifiable cause [3]. The previous estimate of the age of onset for incident dementia was 83.72 ± 5.49 years [4], which is at least 13 years from the beginning of late adulthood (age 65 years). It is unknown if any weight loss observed during this time frame (i.e., prior to the onset of dementia) is a part of the natural progression of dementia. It is plausible that such an observation could be a prognostic factor that appears before the appearance of any phenotypic abnormalities of dementia.

In the early 1990s, Wolf-Klein et al. conducted a study to ascertain the association between Alzheimer’s disease (AD) and weight change pattern [5]. The results of this study revealed a huge difference between the case and control groups; 93% of dementia patients lost weight after the diagnosis, while only 39% of those without dementia lost weight. The study has since become one of the foundations of other investigations that examined the relationship between weight loss and AD. 

Further studies over the past few decades have yet to determine if weight loss is a component or a consequence of AD. Multiple studies have proposed the idea that weight loss can be detected several years prior to a diagnosis of dementia. Barrett-Connor et al. conducted a retrospective study that contained 20 years of follow-up data, and reported that both men and women who were later diagnosed with AD had significant weight loss (≥5 kg) prior their diagnosis [6]. In 2007, Knopman et al. observed a weight loss trend 9 to 10 years prior to the diagnosis of dementia in women [7]. Another study conducted by LeBlanc et al. (2017) observed a greater rate of weight loss (on average additional −0.11 kg/year) over 20 years in women before a diagnosis of mild cognitive impairment (MCI) and dementia [8]. A recent study conducted in 2020 by Lu et al. also suggested that long-term weight loss (≥1.5 kg) can be detected 12 years prior to a diagnosis of disabling dementia in a Japanese population [9]. These studies suggest that weight loss during middle adulthood may serve as a prognostic factor of future cognitive decline. 

On the other hand, there are studies that propose the idea that weight loss is tied to dementia disease progression. Although the difference was not as significant as Wolf-Klein et al.’s finding, in 2004, Wang et al. reported that 54.9% of dementia patients and 14.8% of patients without dementia have lost weight in a case–control study [10]. Stewart et al. reported that people with dementia experienced a higher degree of average yearly weight loss (−0.58 kg/year; 95% CI, −0.53 to −0.19) than people without dementia (−0.22 kg/year; 95% CI, −0.26 to −0.18) [11]. In 2016, Venturelli et al. demonstrated that the AD group lost more weight (−2.5 kg, *p*-value < 0.01) than the control group (−0.4 kg, *p*-value = 0.8) in an one-year study [12]. These studies suggest that weight may be a consequence of AD due to neurological and endocrinological changes associated with the disease.

Additionally, studies also attempted to link genetic factors such as *APOE* genotypes with weight change and cognitive decline. The gene *APOE* encodes for apolipoprotein E, and is shown to be highly associated with AD [13]. There are three polymorphic *APOE* variants (ε2, ε3, and ε4 variants), with the ε4 allele being associated with increased risk of AD, ε3 being neutral, and ε2 being associated with decreased risk of AD [14]. Although the finding was not statistically significant, Stewart et al. reported a lower rate of weight loss in AD cases with APOE ε4 (−0.10 kg/y for APOE ε4 and −0.36 kg/y for non-ε4) [11]. In 2014, Besser et al. observed that weight loss of >4% was associated with faster clinical progression in participants with amnestic mild cognitive impairment (aMCI) and higher disease severity in AD; however, the observation only applied to participants with non-APOE ε4 alleles [15]. These finding suggest that the effect of weight change on cognitive decline may belong to a biological pathway that is independent of *APOE* genetic status. Therefore, we decided to assess other anthropometric genetic factors associated with weight change.

The polygenic risk score (PGS) assesses the genetic variants across the whole genome, and translates the findings into a score that would be meaningful for clinical implementation [16]. There were many clinical factors that could be assessed together with PGS, including, but not limited to, life-time risk of developing a specific disease, differences in anthropometric factors (such as height and weight), and even differences in lifespan. Study suggested that maintaining an optimal BMI was associated with decreased disease risk and increased longevity [17]. There is a possibility that the PGSs associated with BMI and longevity may be tied to weight change in dementia.

It is not an easy task to ascertain the effect of weight change in dementia, and a wide variety of weight assessment methodologies have been used across different studies. For instance, Barrett-Connor et al. considered only a weight change of ≥5 kg meaningful [6]. Lu et al. set the weight change threshold to 1.5 kg [9]. Besser et al. considered yearly weight change of >4% meaningful [15]. Other studies treated all degrees of weight change as meaningful changes [5,7,8,11,12]. Clinicians generally accept 5–10% weight gain/loss as meaningful (either beneficial or harmful) to health [18]. Thus, there was a considerable amount of heterogeneity in the findings, and many of these AD weight loss studies have not reached a consensus. 

In our study, we attempt to elucidate the plausible biological mechanisms of weight changes that distinguish the people who have a memory disorder from people who do not. We postulated that (1) in older adults with a diagnosis of a memory disorder, weight loss would begin starting from middle adulthood (age 45–65 years) and prior to the diagnosis; (2) a person’s BMI from middle adulthood and longitudinal weight change pattern can predict future cognitive decline; (3) weight maintenance can potentially mitigate or lower the risk of dementia for people who are predisposed to genetic factors that are associated with dementia.

## 2. Methods

### 2.1. Cohorts

Study data: The data were obtained from the U.S. Health and Retirement Study (HRS), an on-going longitudinal research study on elder adults ages over 50 that began in 1992 [19,20]. The study populations were selected and the data were extracted from the HRS public survey data [HRS CORE] 1996–2020 public use dataset. We extracted participant demographic information including baseline age, sex, race, ethnicity, weight, height, and data on cancer and memory disorders. The *APOE* genotype data were extracted from the HRS sensitive health data [*APOE* and serotonin transporter alleles] from the restricted dataset. The information on the polygenic risk scores were extracted from the HRS public survey data [polygenic score data (PGS)] public use dataset. All HRS studies are sponsored by the National Institute on Aging (grant number NIA U01AG009740, NIH U01 AGO9740, RC2 AG036495, and RC4 AG039029) and are conducted by the University of Michigan. 

This study used deidentified secondary data from the HRS. They were reviewed by the Clemson University IRB, and were determined to meet the criteria for exempt review (Clemson IRB number: IRB2021-0235). 

### 2.2. Measures

Baseline age: the baseline age was extracted based on the first age entry of each unique participant from the biennial cohorts; 

Sex: the biological sex of each participant was based on the first entry from the survey; 

Race: the race field was categorized into four categories: White/Caucasian, Black/African American, other (Alaskan, Asian, and Pacific Islander), and unknown; 

Hispanic: Hispanic field was recategorized into three categories: yes, no, and unknown; 

Weight measurement: weight measurements were taken for each participant every 2 years between 1996–2020. Weight was measured in pounds (lb), and was converted into kilograms (kg) with the conversion formula: 1 lb = 0.453592 kg; 

Mean biennial percent weight change: the biennial weight change pattern was assessed by obtaining the percent difference between each biannual weight measurement. To account for single missing data between two known weight records, we applied the interpolation method by inputting the average weight. To account for weight fluctuation during the 24 years of follow-up, we took the average of the biennial percent weight change (%WC) of each participant, and obtained the mean biennial %WC. For certain statistical analyses, we categorized the data into three categories: the stable weight, weight loss, and weight gain groups. All biennial %WC between ±0.5 standard deviation (σ) from the mean was categorized as the stable weight group, changes <−0.5 σ were the weight loss group, and >0.5 σ were the weight gain group. For participants with a memory disorder and their matched control group, we also calculated the average biennial %WC prior to diagnosis and after a diagnosis of dementia (simulated year for the control group); 

Height measurement: due to multiple height records, we extracted the median height of each participant for BMI calculation. Height was measured in feet (ft) and inches (in), and was converted into centimeters with the conversion formulas: 1 ft = 30.48 cm and 1 in = 2.54 cm;

Baseline BMI: baseline BMI was calculated with the formula: $\frac{Weight\;\left(\mathrm{kg}\right)}{{\left[Height\;\left(m\right)\right]}^{2}}$. This served as a way to categorize HRS participants into different starting categories. The cutoffs for the BMI categories were: underweight (<18.5), healthy weight (18.5 to 24.9), overweight (25.0 to 29.9), and obese (>30.0). It was interpreted based on the guidelines from the U.S. Centers for Disease Control and Prevention [21];

Cancer diagnosis: the field “Any Cancer Except For Skin Cancer” was used to determine if the participant had a cancer diagnosis; 

Memory disorder diagnosis: memory disorder and memory-related diseases were assessed differently in each survey. The 1996 survey used the medical condition field to confirm an AD diagnosis. In addition to the medical condition field, the 1998–2002 surveys contained the memory-related disease field as dementia flag. The 2004–2008 surveys contained only memory-related disease field as a dementia flag. The 2010–2020 surveys separated the memory-related disease field into two separate categories: ‘Ever had Alzheimer’s Disease’ and ‘Ever had Dementia’ fields. We extracted the year that contained a positive flag in AD or dementia as first year of diagnosis. Participants with answers including ‘Unknown’, ‘Refuse to Answer’, and ‘Don’t Know’ were categorized into non-dementia group. 

### 2.3. Genetic Data

*APOE* genetic data: the dataset included the genotype data of the two *APOE* ε2/ε3/ε4-determining SNPs rs429358 (T/c) and rs7412 (C/t) from 19,193 participants [22]. Based on the genetic data, the participants were assigned with ε2 (rs429358-T; rs7412-t), ε3 (rs429358-T; rs7412-C), and ε4 (rs429358-c; rs7412-c) APOE variants. There were six categories: ε2/ε2, ε2/ε3, ε2/ε4, ε3/ε3, ε3/ε4, and ε4/ε4; 

Polygenic risk scores: two polygenic risk scores (PGS) of interests were selected to determine whether genetic factors could mediate weight change in dementia. The first was the longevity PGS, due to its association with increased life expectancy, which was equivalent to having greater number of protective alleles in the genetic pool. The longevity PGS came from a study conducted by the Cohorts for Heart and Aging Research in Genomic Epidemiology (CHARGE) consortium in 2015. Another PGS of interest was the BMI PGS, due to its association with lifetime weight maintenance. There were two BMI PGSs that were created by the Genetic Investigation of Anthropometric Traits (GIANT) consortium in 2015 and 2018. All PGSs included approximately 2.5 million SNPs for genetic measurements, and the models were adjusted for sex and genetic principal components for population stratification. Additional information on the PGS are described in the HRS PGS documentation report [23]. 

### 2.4. Data Cleaning

Any BMIs less than 10 or greater than 80 were removed from dataset as they were likely due to errors in measurement. Extreme height outliers such as heights less than 100 cm or greater than 220 cm were taken out. Weight data that were less than 20 kg or over 250 kg were also removed. Additionally, we applied the modified path ratio algorithm to remove spurious values from the study [24]. 

We used the following equations:

$$Length\;A=\sqrt{{\left[Weight2\;(\mathrm{kg})-Weight1\;(\mathrm{kg})\right]}^{2}+{\left[Time2\;(\mathrm{yr})-Time1\;(\mathrm{yr})\right]}^{2\;}}+\sqrt{[{Weight3\;\left(\mathrm{kg}\right)-Weight2\;(\mathrm{kg})]}^{2}+{\left[Time3\;\left(\mathrm{yr}\right)-Time2\;\left(\mathrm{yr}\right)\right]}^{2}}$$

$$Length\;B=\sqrt{{\left[Weight3\;(\mathrm{kg})-Weight1\;(\mathrm{kg})\right]}^{2}+{\left[Time3\;(\mathrm{yr})-Time1\;(\mathrm{yr})\right]}^{2\;}}$$

If the ratio of $\frac{Length\;A}{Length\;B}$ exceeds 2.0 (>2.0), the weight value is a spurious value and is removed from the data. 

### 2.5. Exclusion Criteria

We applied the following exclusion criteria for our study: participants with missing age, sex, race, and height data were removed from the study. The ‘Other’ race population accounted for less than 3% of the total study participants; therefore, we only included Black and White populations for the analyses. Study participants with a cancer diagnosis, regardless of the year of diagnosis were removed from the analysis because weight change may be unpredictable in this subgroup. To ensure that there were consecutive weight entries for data interpolation, we removed any participants that had less than six weight measurements over the span of the study. Lastly, we removed the Hispanic population from the analyses; they accounted for less than 5% of the data after we applied all other exclusion criteria, and genetic admixture may affect models for genotype analyses [25,26]. 

### 2.6. Age–Race–Sex-Matched Control Selection for Nested Case–Control Study

We used baseline age (±1 year), race, sex, and year of enrollment as anchor to pull age–sex–race-matched controls from the cohort to match for the cases of dementia for the nested case–control study portion of the investigation. A total of 1509 cases were able to pair with exact matches, and an additional 27 cases with age ±1 years. A simulated memory disorder diagnosis year was assigned based on each control’s memory disorder case counterpart. We used it to compute for a simulated pre- and post-diagnosis mean biennial %WC for the control group. 

### 2.7. Statistics

Independent samples *t*-test: an independent samples *t*-test was performed to compare the degree of weight change between the disease and non-disease group. Levene’s test was used to assess the equality of variances for the variables analyzed in the *t*-test; 

Analysis of covariance: the effects of memory disorder, race, and sex on the mean % biennial weight change were further tested with analysis of covariance (ANCOVA), and the model was adjusted with baseline BMI and baseline age of the participants. The following analyses were conducted on the cases and their respective age–race–sex-matched controls; 

Binary logistic regression: The regression model was used to ascertain the predictability of baseline BMI, age, race, sex, and mean biennial %WC on a diagnosis of a memory disorder. The same statistic model was also applied to determine the predictability of additional genetic factors including *APOE* haplotype, longevity PGS, and BMI PGSs on the nested case–control portion of the cohort that contained only dementia cases and their age–sex-race matched controls; 

Paired sample *t*-test: a paired samples *t*-test was performed to compare the means of the biennial %WC before and after a diagnosis of a memory disorder. The analysis was also performed on the age–race–sex-matched controls with a simulated year of diagnosis. Participants without a biennial %WC measure for either pre- or post-diagnosis were removed from the analysis; 

Chi-square automatic interaction detection analysis: the hierarchy between the variables that could determine the incidence of dementia were tested with the chi-square automatic interaction detection (CHAID) analysis. This was originally a decision tree model that was commonly used in economics research that used automated algorithms to determine the most significant covariate and hierarchically organized samples into sub-categories based on the interactions of the independent variables with the dependent variable [27]. In this analysis, we used this method to ascertain the relationship between the important covariates. We only included the variables that were significantly associated with a memory disorder diagnosis in the binary regression model. It was performed only on the secondary cohort, and the participants missing data for *APOE* genotype were removed from the analysis. 

All statistical analyses were conducted with IBM SPSS Statistics for Windows, version 20.0 [28]. We used Microsoft Office Professional Plus 2019 to create and annotate figures [29]. 

## 3. Results

### 3.1. Participant Demographic Characteristics

The full demographic information is depicted in Table 1. A total of 13,123 samples were selected from 39,015 participants for the study after applying all of the exclusion criteria. The mean baseline age of the population was 58.74 years, 59.2% were female, and 78.6% were White. There were 1579 participants (12.0%) who developed a new incident diagnosis of a memory disorder (AD or dementia) during the follow-up period, the mean age of diagnosis was 77.26 years, and the subgroup’s baseline age was 65.33 years. The mean baseline BMI for the population with a memory disorder was 27.53 BMI points (SD = 5.53) and 28.01 BMI points (SD = 5.62) for population without a memory disorder [*p*-value = 0.001 (95% CI: −0.77, 0.19), *t*-test].

### 3.2. Weight Change Patterns in Dementia 

**Figure 1:**
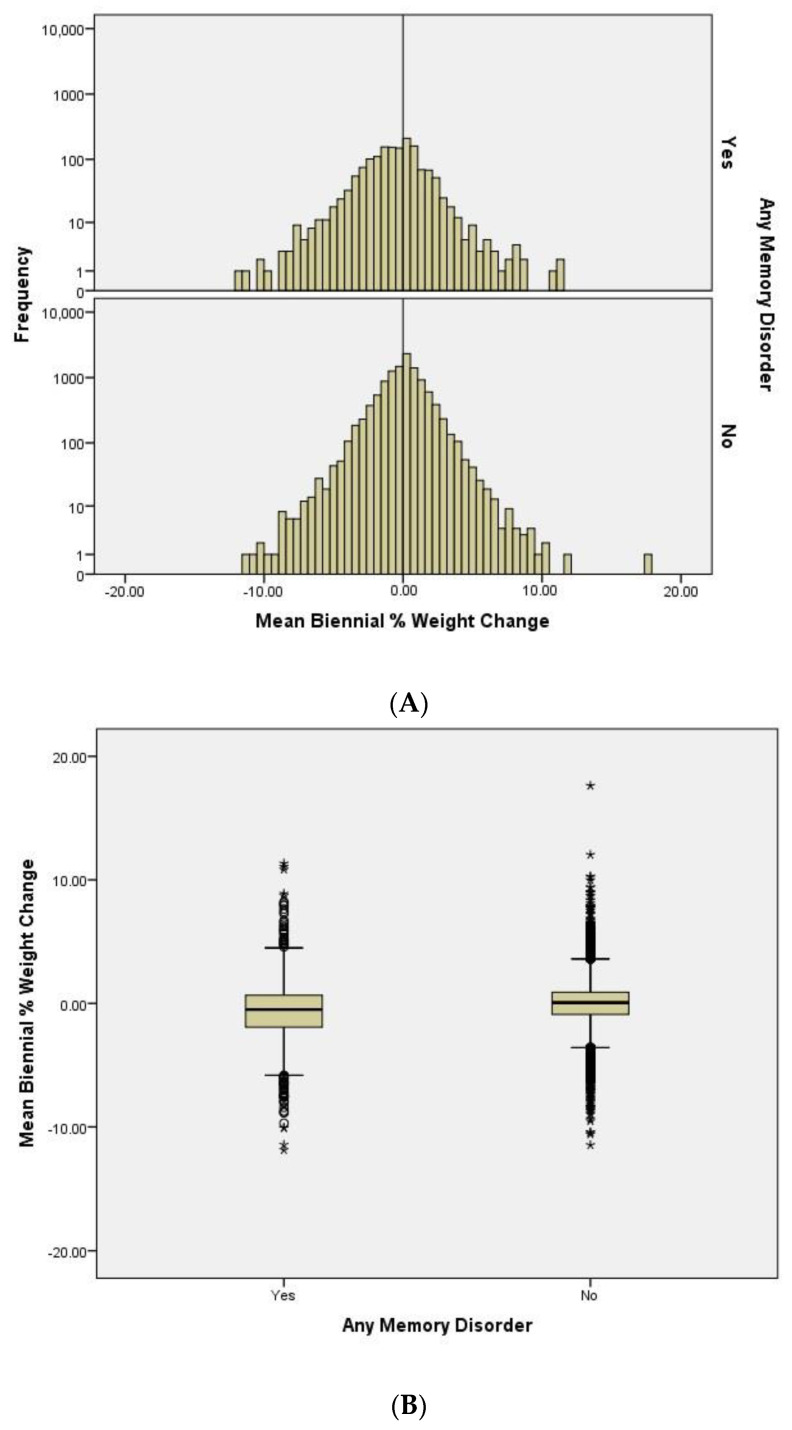

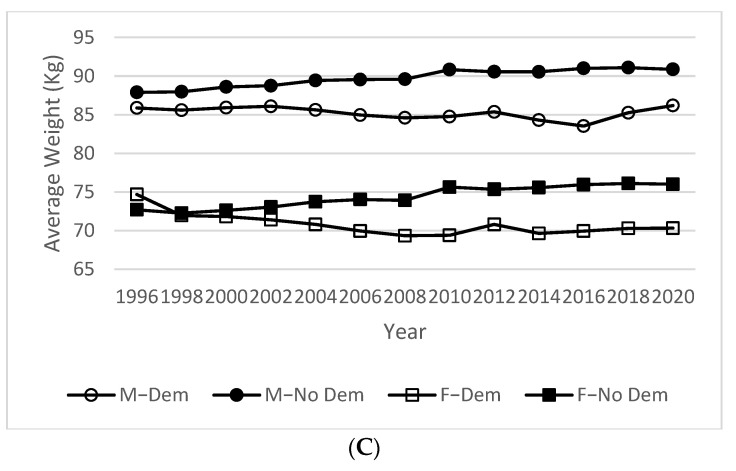
**Weight change patterns of participants with and without a memory disorder**. Overview of the weight patterns between individuals with and without a diagnosis of a memory disorder. (**A**) A histogram that illustrates the distribution of mean biennial percent weight change during the 24 years follow-up period; frequency in log_10_ scale. (**B**) The overall mean and median of the mean biennial percent weight change between case and control group; sample means *p*-value < 0.0001 and the * were outliers. (**C**) The biyearly average weight trend (in kilograms) of the study participants separated by sex and dementia status; male in circular markers, female in square markers, dementia colored in white, and non-dementia colored in black. % = percent; kg = kilograms; M-Dem = male with dementia; M-No Dem = male without dementia; F-Dem = female with dementia; F-No Dem = female without dementia.

We compared the distributions of the mean biennial percentage weight change between participants with and without dementia (Figure 1A). We observe that the population is approximately normally distributed in the non-dementia group. The histogram also shows that the distribution of the population with a memory disorder is left-shifted to more weight loss. This suggests that there are more people losing weight than gaining weight in the dementia group. 

To determine the differences in the weight change pattern between the dementia and non-dementia group, we plotted the mean biennial %WC and compared their means (Figure 1B). As expected, we observe significant differences [*p*-value < 0.0001 (95% CI: −0.77, −0.52), *t*-test] between the two groups. Participants with a memory disorder show a higher degree of weight loss (mean = −0.63, SD = 2.42) compared to people without a memory disorder (mean = 0.013, SD = 1.80) during the follow-up timeframe.

Figure 1C illustrates the biennial average weight data (in kg) of the participants separated by sex and dementia status. Both male and female populations with dementia have lower average weight during the 24 years of follow-up. When compared to the female counterpart, male participants have fairly stable weight trends, with the non-dementia group gaining slightly more weight. Female participants have very different trends of weight change between the two groups; the participants with a memory disorder on average lose approximately 7% of body weight (percent difference between final weight and baseline weight), and the non-dementia group on average gain about 5% of body weight. 

Table 2 summarizes the interaction effect statistics of memory disorder, sex, and race on biennial %WC pattern. In general, females experience higher degree of weight loss than males, regardless of disease status. The biggest difference in the mean %WC is observed in white females (−0.299) and white males (−0.066). Females with a diagnosis of dementia experience the greatest amount of mean biennial % weight loss during the follow-up period, with an average of −0.317% (SE = 0.067, 95% CI: −0.449, −0.185).

### 3.3. Weight Loss as a Predictor of a Memory Disorder

Due to the sample size differences between the dementia (n = 1579) and non-dementia (n = 11,544) groups, we determined that it was necessary to apply matching techniques to improve statistical precision. We created a nested case–control study cohort with age–race–sex-matched controls selected using the criteria described in the methodology section. Out of the 1579 cases, 1536 of them were able to pair with an age–race–sex-matched control. The sample demographics of both groups are summarized in Table 3. 

The mean baseline demographic characteristics of baseline age, race, sex, BMI, and age at diagnosis are similar for the cases and controls groups, (*p* > 0.05), as expected due to matching. The mean biennial %WC of all participants is −0.532 (SD = 2.11); the mean and standard deviation of %WC was used to determine each participant’s weight change category, as described in the methodology section. 

To ascertain whether weight loss was a predictor of a memory disorder, we conducted an analysis with binary logistic regression. A total of 3115 participants (1579 cases and 1536 controls) were included in the regression model; the results are depicted in Table 4. After adjusting for the matching factors of baseline age, sex, and race, the biennial %WC is a significant predictor of a memory disorder. Participants in the weight loss group (<−0.5 SD from the mean) have two-fold (*p*-value < 0.01; 95% CI: 1.672, 2.417) higher odds of developing a memory disorder than participants in the stable weight group, whereas participants from the weight gain group (>0.5 SD from the mean) are 43.4% (*p*-value < 0.01; 95% CI: 1.208, 1.704) more likely to have a diagnosis of a memory disorder. The variable baseline age was a continuous variable and other variables were categorical. Although the mean biennial %WC is a significant predictor of a memory disorder, the regression model has poor overall predictive ability. The predictability of the overall model is 56.4% and the pseudo-R^2^ estimated with the Cox–Snell likelihood model is 0.021.

### 3.4. Weight Change Pattern before and after Dementia Diagnosis

We next compared the differences in the weight change pattern prior to and after a diagnosis of a memory disorder (Table 5). Some of the participants were removed from the analysis due to insufficient weight measurements to calculate a pre-diagnosis and post-diagnosis biennial %WC. There were 1088 cases and 1004 matched controls included in the analyses. 

In both case and the matched control groups, we observe an increased degree of weight loss after a diagnosis of dementia. The mean %WC of the case group is −0.29 (SD = 4.76) pre-diagnosis and is −0.71 (SD = 8.12) post-diagnosis. However, the result is not statistically significant [mean difference = 0.42, *p*-value = 0.167 (95% CI: −0.17, 1.01), paired *t*-test]. The mean %WC of the age–race–sex-matched control group is 0.19 (SD = 2.72) pre-diagnosis and is −1.05 (SD = 4.39) post-diagnosis. The difference observed in the control group is statistically significant [mean difference = 1.24, *p*-value < 0.0001 (95% CI: 0.90, 1.57), paired *t*-test]. 

Additionally, we tested for the difference of the pre- and post-dementia diagnosis weight change pattern between the case and control groups. Statistical analysis reveals a significant difference in the mean biennial %WC between the case (−0.29) and control (0.19) groups before a diagnosis of a memory disorder [mean difference = 0.48, *p*-value = 0.005 (95% CI: 0.15, 0.80), *t*-test], while the differences in the mean biennial %WC between the case (−0.71) and control (−1.05) groups after a dementia diagnosis are not significantly different [mean = −0.35, *p*-value = 0.22 (95% CI: −0.90, 0.21), *t*-test]. These results suggest that a middle adulthood weight change pattern may be different for people who eventually develop dementia. 

### 3.5. Genetic Factors and Weight Change Patterns in Dementia

To determine if genetic variables would affect the predictability of biennial %WC for a memory disorder, we conducted another regression analysis with *APOE*, longevity PGS, and both BMI PGSs (Table 6). The biennial %WC is still significantly associated with the diagnosis of a memory disorder. Participants in the weight loss group are 110.2% (OR 2.102, *p*-value < 0.001; 95% CI: 1.673, 2.641) more likely to have a diagnosis of a memory disorder, and the weight gain group (>0.5 SD from the mean) are 41.6% (OR 1.416, *p*-value < 0.001; 95% CI: 1.143, 1.754) less likely to have a diagnosis. Baseline age is a significant (*p*-value = 0.026; 95% CI: 0.979, 0.999) covariate in this model. When compared to the neutral ε3/ε3 population, the ε3/ε4 (OR = 2.13, *p*-value < 0.001, 95% CI: 1.635, 2.485) and ε4/ε4 (OR = 3.15, *p*-value < 0.001, 95% CI: 2.622, 9.316) *APOE* groups are significantly correlated with a higher risk of developing a memory disorder. All of the other *APOE* variants are correlated with lower risk, but are not significant: ε2/ε2 (OR = 0.27, *p*-value = 0.189, 95% CI: 0.028, 2.023); ε2/ε3 (OR = 0.74, *p*-value = 0.915, 95% CI: 0.747, 1.299); ε2/ε4 (OR = 0.85, *p*-value = 0.309, 95% CI: 0.444, 1.293). All of the remaining covariates including race, longevity PGS, and BMI PGSs are not significantly correlated with a diagnosis of a memory disorder. All the PGSs are continuous variables. Similar to the previous model (Table 4), this model has low predictive ability. The predictability of the overall model is 61.2%, and the pseudo-R^2^ estimated with the Cox–Snell likelihood model is 0.061.

**Figure 2:**
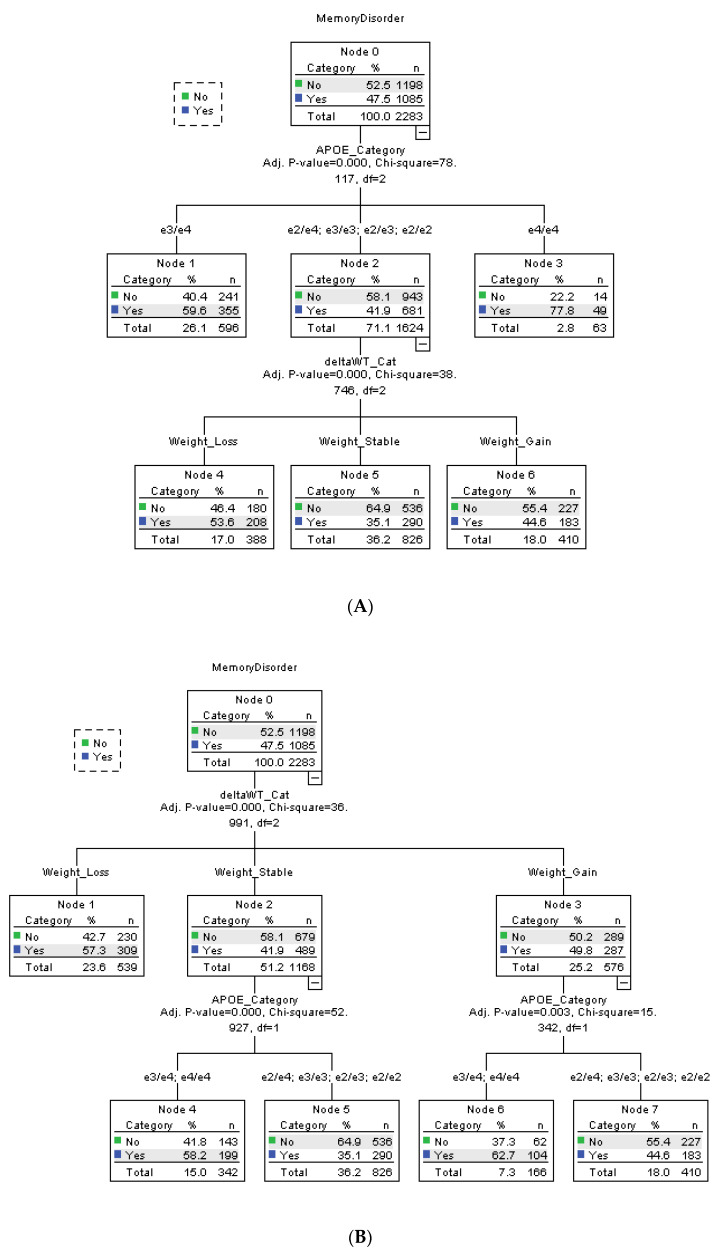
CHAID analysis of *APOE*, sex, and weight change pattern. deltaWT_Cat = mean biennial % weight change category. CHAID decision tree analysis was used to determine the hierarchical order of the risk factors that were associated with incidence of a memory disorder. Factors assessed included APOE haplotypes, sex, and mean biennial % weight change. (**A**) CHAID analysis with automated algorithm. (**B**) CHAID analysis with ‘forced first variable’ method to directly assign mean biennial % weight change as highest order risk factor.

The hierarchical relationship between the statistically significant risk factors (*APOE*, mean biennial % weight change, and sex) were ascertained through CHAID analysis (Figure 2). Using automated algorithm, the statistical software automatically assigned and ranked each risk factors; the result is depicted in Figure 2A. APOE ε4/ε4 and ε3/ε4 are the main risk factor of a memory disorder, with 350% (ε4/ε4) and 47.3% (ε3/ε4) more participants within the subgroup having a dementia diagnosis. The remaining APOE variants ε2/ε2, ε2/ε3, ε2/ε4, and ε3/ε3 are classified together as the lower risk group. Among the lower risk APOE group, participants in the weight loss group are 15.6% more likely to have a dementia diagnosis, independent of *APOE* ε4 status. 

To confirm whether mean biennial %WC is a risk factor independent of *APOE*, we reran the CHAID analysis and assigned the weight variable as highest order risk factor (Figure 2B). The result suggests that participants who lose weight are 34.3% more likely to develop the disease, regardless of *APOE* SNPs. In stable weight and weight gain groups, participants with APOE ε4/ε4 and ε3/ε4 are 39.2% (stable weight) and 67.7% (weight gain) more likely to have a memory disorder. 

## 4. Discussions

Our findings suggest that, in general, there are more people in the dementia group who lose weight compared to people without a diagnosis of dementia. On average, a person with a memory disorder experiences −0.63% of biennial % weight loss during the 24 years of follow-up. For example, for a person that weighed 150 lbs, a 0.63% weight loss would be equivalent to losing 0.945 lb per 2 years, or, cumulatively, 11.34 lb in 24 years. However, this number is not considered as clinically meaningful unless the cumulative loss exceeds that of 5% [18]. It is recommended that future studies should account for duration of years of follow-up when drawing a cut-off point for meaningful weight changes. For instance, shorter studies may be required to set a less stringent criteria to capture meaningful weight trajectories in participants with dementia. This could potentially prevent studies from unknowingly excluding meaningful data for their studies. 

There is a significant interaction effect of sex and diagnosis of a memory disorder. The female population with a diagnosis of dementia experiences higher mean biennial % weight loss when compared to their male counterparts and people without dementia. This finding is consistent with Knopman et al.’s and Le Blanc et al.’s findings of higher degree of mid-life weight loss observed in female populations with memory disorders [7,8]. Additionally, even though there is no interaction effect between race and dementia diagnosis, the effect is significant between race and sex. Females from white population have a higher tendency to lose more weight than black females, and males of any races. 

Our study demonstrates that weight loss precedes a diagnosis of a memory disorder. On average, participants with dementia experience −0.29% biennial weight loss for multiple years prior to the clinical diagnosis, many of whom begin weight loss in middle adulthood. This pattern is not observed in the age–sex–race-matched control groups, where people gain an average of 0.24% of body weight biennially around a similar age. There is a minimum of a 2 year gap in between the weight measurement taken at the year of diagnosis and the first weight record that precedes the diagnosis; and up to 20 years of pre-dementia weight trajectories are taken into calculation to account for weight fluctuations. Therefore, it is unlikely that the observation of pre-dementia weight loss is an artifact of one-year accelerated weight loss due to preclinical cognitive decline [30]. This also suggests that our finding aligns with previously conducted research that suggests that a higher degree of weight loss can be observed in patients several years before a diagnosis of dementia [7,8,9,11,30]. Our results are also in agreement with two recent publications using different analytical approaches that identified pre-diagnosis weight changes as a predictor of dementia [31,32].

Although earlier findings demonstrate that dementia is a causal factor of weight loss [33,34], our study suggests otherwise. There are insignificant differences in weight change patterns between the cases and controls after a dementia diagnosis (simulated diagnosis for control group). Analyses of the matched controls reveals that even without a diagnosis of a memory disorder, participants eventually lose weight as they reach a certain age. In fact, the participants from the control group have a slightly greater difference in their pre- and post-diagnosis (simulated) weight change pattern. Nevertheless, based on our findings, weight loss is still a strong prognostic factor and predictor of dementia. 

Our result confirms that the mean biennial %WC loss is a strong predictor of dementia; the odds ratio is almost equivalent to that of having a single APOE ε4 allele (i.e., ε3/ε4 populations). Participants categorized in the weight loss group are more likely to develop a memory disorder, while people who have stable weight are less likely to develop the disease. There are also considerable numbers of people with memory disorders in the weight gain group, which suggests that gaining weight is not protective against dementia. This finding is in line with a previous study that reported that even though there was a weight loss trend in dementia patients, many of the study population actually gained 2.5  ±  2.3 kg in weight [35]. 

The observation of weight loss in dementia may be more complicated than it appears to be. Previous studies suggested that healthy BMI was a predictor of disease-free life expectancy between ages 50 and 75 [36], and a BMI ranging from 20.0 to 24.9 was associated with decreased all-cause mortality [37]. In order to maintain a healthy BMI, it would require proper weight maintenance either through weight gain, weight loss, or maintaining a stable weight. Therefore, in theory, BMI and anthropometric genetic risk factors associated with BMI and longevity should be linked to weight loss in dementia. However, our study does not find any strong association between BMI, BMI PGS, and longevity PGS with incidences of dementia. When we account for the *APOE* haplotype (the strongest genetic risk factor associated with AD) in the model, our analyses reveal that having the APOE ε4/ε4 variant and being categorized in the weight loss group are both important risk factors of dementia. 

When the participants are clustered based by their APOE variant, people with APOE ε3/ε4 and ε4/ε4 have the highest dementia risk. For people with the other APOE variants (ε2/ε2, ε2/ε3, and ε2/ε4, and ε3/ε3), people who lose weight have the highest risk of developing dementia. If the participants are categorized based on their biennial weight change pattern first, the analysis suggests that weight loss is a risk factor completely independent of *APOE* variants. Within the stable weight and weight gain groups, APOE ε3/ε4 and ε4/ε4 variants remain the strongest risk factor for dementia. These findings are similar to those of previous studies that suggest that the ε4 allele could potentially mask the effect of weight change in dementia, and the effect of weight loss is strongest in non-ε4 carriers [14,15]. 

Our study has several strengths, which includes a large sample size of elderly adults, with 24 years of longitudinal data for the study of incident cases of dementia. The availability of *APOE* genotype data allowed us to ensure that our observation was not an artifact of known genetic risk factors. Additionally, pre-calculated polygenic risk scores of BMI and longevity provided us with a way to ascertain the effect of a collection of genes on weight change in dementia. There were a few limitations for our study; our analysis focused on weight change, but the available data in the HRS could not elucidate whether the observed changes in weight over time were intentional or unintentional. To normalize the effect of weight change between each individual, we assessed the biennial percent weight change rather than absolute or other measures of weight change and used ± 0.5 standard deviation (σ) as a cutoff point to categorize %WC into appropriate categories. Due to this, we did not attempt to classify a specific weight or percent change cut-off as meaningful weight change for the study. Information on the specific type of dementia, stage of disease, and neuroimaging results were not available in the core data and could not be assessed in this analysis. Finally, future studies would benefit from the inclusion of more diverse populations and an assessment of additional comorbid health conditions such as diabetes and cardiovascular disease. 

## 5. Conclusions

Our study elucidates the importance of weight change in dementia, and reveals that long-term weight loss, and especially weight loss during middle adulthood (prior to a diagnosis of dementia) are closely associated with the development of a memory disorder. In our research, we find that weight change, even subtle yearly weight loss, is a strong predictor of dementia. When compared to weight loss and weight gain groups, people who are able to maintain a stable weight during their middle-to-late adulthood are less likely to develop a memory disorder. From our study, we conclude that the biological mechanism of weight loss in dementia is independent of APOE variants and other genetic factors that are linked to human longevity and BMI maintenance. Future research on the effect of weight change in memory disorders should take account of the *APOE* genotype of the participants, and the duration of follow-up. This will improve the overall accuracy, and increase between-study homogeneity to help identify potential biological mechanisms that contribute to weight loss that precedes dementia. Weight loss can potentially become an important prognostic factor for clinicians to identify high-risk individuals for clinical interventions. 

## Figures and Tables

**Table 1:** Sample characteristics of the cohort to investigate weight change and memory disorder; Health and Retirement Study.

Field	Male	Female	Total
**Sample size (n)**	5356 (40.8%)	7767 (59.2%)	13,123
**Racial breakdown (n)**			
White	4386 (81.9%)	5927 (76.3%)	10,313 (78.6%)
Black	970 (18.1%)	1840 (23.7%)	2810 (21.4%)
**Mean baseline age (Yr)**			
All	58.79	58.70	58.74
With memory disorder	63.99	66.16	65.33
Without memory disorder	58.13	57.63	57.84
**Any memory disorder diagnosis (n)**	603 (11.3%)	976 (12.6%)	1579 (12.0%)
**Mean age of diagnosis for memory Disorder (Yr)**	75.40	78.41	77.26
**Mean baseline BMI (BMI)**			
With memory disorder	27.50 (SD 4.75)	27.55 (SD 5.96)	27.53 (SD 5.53)
Without memory disorder	28.14 (SD 4.78)	27.92 (SD 6.14)	28.01 (SD 5.62)

n = sample size; Yr = year; BMI = body mass index; SD = standard deviation.

**Table 2:** Comparison of weight change pattern among participants of different disease status, race, and sex (adjusted with baseline age and BMI).

Compared Samples	n	Adjusted Mean ^a^ (SE; 95% CI)	*p*−Value ^b^
**With memory disorder**			
Male	603	−0.23 (SE 0.08; −0.40–−0.07)	0.044 *
Female	976	−0.32 (SE 0.07; −0.45–−0.07)	
**Without memory disorder**			
Male	4753	−0.14 (SE 0.03; −0.20–−0.07)	
Female	6791	−0.23 (SE 0.03; −0.07–0.03)	
			
**With memory disorder**			
White	1272	−0.20 (SE 0.05; −0.30–−0.09)	0.676
Black	307	−0.35 (SE 0.10; −0.56–−0.15)	
**Without memory disorder**			
White	9041	−0.03 (SE 0.02; −0.06–−0.01)	
Black	2503	−0.13 (SE 0.04; −0.21–−0.06)	
			
**Male**			
White	4386	−0.07 (SE 0.04; −0.15–0.15)	0.010 *
Black	970	−0.16 (SE 0.03; −0.22–−0.09)	
**Female**			
White	5927	−0.30 (SE 0.08; −0.45–0.15)	
Black.	1840	−0.18 (SE 0.06; −0.30–−0.07)	

Dependent variable: mean biennial % weight change. R-squared = 0.118 (adjusted R-squared = 0.117). a = model adjusted with covariates: baseline BMI = 27.95 and baseline age = 58.74. b = *p*-value from ANCOVA analysis. * = statistically significant (*p*-value < 0.05). n = sample size; SE = standard error.

**Table 3:** Sample characteristics of the nested case–control cohort.

Field	Case	Control
**Sample size (n)**		
Female	976 (61.8%)	951 (61.9%)
Male	603 (38.2%)	585 (38.1%)
**Racial breakdown (n)**		
White	1272 (80.6%)	1256 (81.8%)
Black	307 (19.4%)	280 (18.2%)
**Mean baseline age (Yr)**	65.34	65.18
**Mean age of diagnosis for memory disorder (Yr)**		
Actual	77.43	-
Simulated	-	77.34
**Mean baseline BMI (BMI)**	27.53 (SD 5.53)	27.35 (SD 5.08)
**Mean % biennial weight change**	−0.63 (SD 2.42)	−0.42 (SD 1.78)

n = sample size; Yr = year; BMI = body mass index; SD = standard deviation.

**Table 4:** Multiple regression analyses of the nested case–control.

Memory Disorder	OR (95% CI)	*p*-Value
**Biennial % weight change**		
Weight stable	1	1.36 × 10^−13^ *
Weight loss	2.01 (1.67–2.42)	1.06 × 10^−13^ *
Weight gain	1.43 (1.21–1.70)	3.99 × 10^−5^ *
**Baseline BMI**		
Healthy	1	0.170
Underweight	0.96 (0.46–1.99)	0.904
Overweight	0.83 (0.69–0.98)	0.026
Obese	0.89 (0.74–1.08)	0.226
**Sex**		
Male	1	
Female	0.93 (0.80–1.08)	0.330
**Race**		
White	1	
Black	1.07 (0.89–1.28)	0.494
**Baseline Age**	0.99 (0.99–1.00)	0.372

n = 3115. Predictability of the model: 56.4%; Cox–Snell R-square: 0.021. * = statistically significant (*p*-value < 0.01).

**Table 5:** Weight change pattern before and after a memory disorder diagnosis.

Compared Samples	Mean (SD)	Mean Differences (SD or SE; 95% CI)	*p*−Value
**Cases**			
Pre−DX mean biennial %WC	−0.29 (4.76)	0.42 (SD 9.97;	0.167
Post−DX mean biennial %WC	−0.71 (8.12)	−0.18–1.01)	
			
**Controls**			
Pre−DX mean biennial %WC	0.19 (2.72)	1.24 (SD 5.41;	7.76 × 10^−13^ *
Post−DX mean biennial %WC	−1.05 (4.39)	0.90–1.57)	
			
**Pre−DX mean biennial %WC**			
Cases	−0.29 (4.76)	0.48 (SE 5.41;	0.005 *
Controls	0.19 (2.72)	0.15–0.80)	
			
**Post−DX mean biennial %WC**			
Cases	−0.71 (8.12)	−0.35 (SE 0.17;	0.222
Controls	−1.05 (4.39)	−0.90–0.21)	

n = 1088 for cases; n = 1004 for controls. * = statistically significant (*p*-value < 0.01). %WC = percentage weight change; DX = diagnosis; SD = standard deviation; SE = standard error difference.

**Table 6:** Multiple regression analyses with *APOE* and polygenic risk scores.

Memory Disorder	OR (95% CI)	*p*-Value
**Biennial % weight change**		
Weight stable	1	5.31 × 10^−10^ **
Weight loss	2.10 (1.67–2.64)	1.75 × 10^−10^ **
Weight gain	1.42 (1.14–1.75)	0.001 **
**Baseline BMI**		
Healthy	1	0.391
Underweight	0.41 (0.13–1.34)	0.141
Overweight	0.90 (0.73–1.10)	0.299
Obese	0.93 (0.73–1.18)	0.545
**Sex**		
Male	1	
Female	0.86 (0.72–1.03)	0.108
**Race**		
White	1	
Black	0.88 (0.69–1.12)	0.300
**Baseline age**	0.99 (0.98–0.99)	0.026 *
***APOE***		
ε3/ε3	1	7.90 × 10^−14^ **
ε2/ε2	0.27 (0.03–2.02)	0.189
ε2/ε3	0.94 (0.75–1.30)	0.915
ε2/ε4	0.85 (0.44–1.29)	0.309
ε3/ε4	2.13 (1.64–2.49)	5.29 × 10^−11^ **
ε4/ε4	3.15 (2.62–9.32)	7.79 × 10^−7^ **
**Longevity PGS**	0.95 (0.87–1.04)	0.262
**BMI PGS 15′**	0.95 (0.84–1.08)	0.448
**BMI PGS 18′**	0.96 (0.84–1.10)	0.600

n = 2283. Predictability of the model: 61.2%; Cox–Snell R-square: 0.061. * = statistically significant (*p*-value < 0.05). ** = highly statistically significant (*p*-value < 0.01).

## Data Availability

The survey data and polygenic score data from the Health and Retirement Study are publicly available through https://hrs.isr.umich.edu/, (accessed on 23 February 2022). The APOE data from APOE and serotonin transporter alleles are part of a restricted dataset, and are available upon filing a data use agreement form.

## References

[1] Lee J.-W., Yoo J.-H., Shin J.-Y., Keum J.-H. (2017). Weight Loss and All-Cause Mortality in the Elderly: A Meta-Analysis. Korean J. Fam. Pract..

[2] De Stefani F.D.C., Pietraroia P.S., Fernandes-Silva M.M., Faria-Neto J., Baena C.P. (2018). Observational Evidence for Unintentional Weight Loss in All-Cause Mortality and Major Cardiovascular Events: A Systematic Review and Meta-Analysis. Sci. Rep..

[3] Gaddey H.L., Holder K. (2014). Unintentional weight loss in older adults. Am. Fam. Physician.

[4] Plassman B.L., Langa K.M., McCammon R.J., Fisher G.G., Potter G.G., Burke J.R., Steffens D.C., Foster N.L., Giordani B., Unverzagt F.W. (2011). Incidence of dementia and cognitive impairment, not dementia in the United States. Ann. Neurol..

[5] Wolf-Klein G.P., Silverstone F.A., Levy A.P. (1992). Nutritional patterns and weight change in Alzheimer patients. Int. Psychogeriatr..

[6] Barrett-Connor E., Edelstein S.L., Corey-Bloom J., Wiederholt W.C. (1996). Weight loss precedes dementia in community-dwelling older adults. J. Am. Geriatr. Soc..

[7] Knopman D.S., Edland S.D., Cha R.H., Petersen R.C., Rocca W.A. (2007). Incident dementia in women is preceded by weight loss by at least a decade. Neurology.

[8] LeBlanc E.S., Rizzo J.H., Pedula K.L., Yaffe K., Ensrud K.E., Cauley J., Cawthon P.M., Cummings S., Hillier T.A., Study of Osteoporotic Fractures Research G. (2017). Weight Trajectory over 20 Years and Likelihood of Mild Cognitive Impairment or Dementia Among Older Women. J. Am. Geriatr. Soc..

[9] Lu Y., Sugawara Y., Matsuyama S., Tsuji I. (2020). Association between Long-term Weight Change since Midlife and Risk of Incident Disabling Dementia among Elderly Japanese: The Ohsaki Cohort 2006 Study. J. Epidemiol..

[10] Wang P.N., Yang C.L., Lin K.N., Chen W.T., Chwang L.C., Liu H.C. (2004). Weight loss, nutritional status and physical activity in patients with Alzheimer’s disease. A controlled study. J. Neurol..

[11] Stewart R., Masaki K., Xue Q.L., Peila R., Petrovitch H., White L.R., Launer L.J. (2005). A 32-year prospective study of change in body weight and incident dementia: The Honolulu-Asia Aging Study. Arch. Neurol..

[12] Venturelli M., Ce E., Limonta E., Muti E., Scarsini R., Brasioli A., Schena F., Esposito F. (2016). Possible Predictors of Involuntary Weight Loss in Patients with Alzheimer’s Disease. PLoS ONE.

[13] Alzheimer’s Association (2020). 2020 Alzheimer’s Disease Facts and Figures. Alzheimer’s Dement. J. Alzheimer’s Assoc..

[14] Liu C.C., Liu C.C., Kanekiyo T., Xu H., Bu G. (2013). Apolipoprotein E and Alzheimer disease: Risk, mechanisms and therapy. Nat. Rev. Neurol..

[15] Besser L.M., Gill D.P., Monsell S.E., Brenowitz W., Meranus D.H., Kukull W., Gustafson D.R. (2014). Body mass index, weight change, and clinical progression in mild cognitive impairment and Alzheimer disease. Alzheimer Dis. Assoc. Disord..

[16] Lewis C.M., Vassos E. (2020). Polygenic risk scores: From research tools to clinical instruments. Genome Med..

[17] Fontana L., Hu F.B. (2014). Optimal body weight for health and longevity: Bridging basic, clinical, and population research. Aging Cell.

[18] Jensen M.D., Ryan D.H., Apovian C.M., Ard J.D., Comuzzie A.G., Donato K.A., Hu F.B., Hubbard V.S., Jakicic J.M., Kushner R.F. (2014). 2013 AHA/ACC/TOS guideline for the management of overweight and obesity in adults: A report of the American College of Cardiology/American Heart Association Task Force on Practice Guidelines and The Obesity Society. J. Am. Coll. Cardiol..

[19] Sonnega A., Faul J.D., Ofstedal M.B., Langa K.M., Phillips J.W., Weir D.R. (2014). Cohort Profile: The Health and Retirement Study (HRS). Int. J. Epidemiol..

[20] Justor F.T., Suzman R. (1995). An Overview of the Health and Retirement Study. J. Hum. Resour..

[21] Centers for Disease Control and Prevention CDC. Healthy Weight, Nutrition, and Physical Activity: About Adult BMI. https://www.cdc.gov/healthyweight/assessing/bmi/adult_bmi/index.html.

[22] HRS Health and Retirement Study HRS: APOE and Serotonin Transporter Alleles Data Descriptions. https://hrsdata.isr.umich.edu/sites/default/files/documentation/data-descriptions/1629310147/File-Description-for-APOE-and-Serotonin.pdf.

[23] Ware E., Gard A., Schmitz L., Faul J. (2021). HRS Polygenic Scores—Release 4.3: 2006–2012 Genetic Data.

[24] Chen S., Banks W.A., Sheffrin M., Bryson W., Black M., Thielke S.M. (2018). Identifying and categorizing spurious weight data in electronic medical records. Am. J. Clin. Nutr..

[25] Bryc K., Auton A., Nelson M.R., Oksenberg J.R., Hauser S.L., Williams S., Froment A., Bodo J.M., Wambebe C., Tishkoff S.A. (2010). Genome-wide patterns of population structure and admixture in West Africans and African Americans. Proc. Natl. Acad. Sci. USA.

[26] Healy M.E., Hill D., Berwick M., Edgar H., Gross J., Hunley K. (2017). Social-group identity and population substructure in admixed populations in New Mexico and Latin America. PLoS ONE.

[27] Milanović M., Stamenković M. (2017). CHAID Decision Tree: Methodological Frame and Application. Econ. Themes.

[28] (2012). IBM SPSS Statistics for Windows, 20.0.

[29] He Y., Li C., Yang Y., Li Y., Wang Y., Yang H., Jin T., Chen S. (2016). Meta-analysis of the rs2075650 polymorphism and risk of Alzheimer disease. Aging Clin. Exp. Res..

[30] Johnson D.K., Wilkins C.H., Morris J.C. (2006). Accelerated weight loss may precede diagnosis in Alzheimer disease. Arch. Neurol..

[31] Shen J., Chen H., Zhou T., Zhang S., Huang L., Lv X., Ma Y., Zheng Y., Yuan C. (2022). Long-term weight change and its temporal relation to later-life dementia in the Health and Retirement Study. J. Clin. Endocrinol. Metab..

[32] Chen H., Zhou T., Guo J., Ji J.S., Huang L., Xu W., Zuo G., Lv X., Zheng Y., Hofman A. (2021). Association of long-term body weight variability with dementia: A prospective study. J. Gerontol. A Biol. Sci. Med. Sci..

[33] Kaneshiro E., Hazama S., Takahata T., Kadoya Y., Tagami S., Yamaguchi K. (1993). Factors for weight loss in patients with senile dementia. Nihon Ronen Igakkai Zasshi.

[34] Shatenstein B., Kergoat M.J., Nadon S. (2001). Anthropometric changes over 5 years in elderly Canadians by age, gender, and cognitive status. J. Gerontol. A Biol. Sci. Med. Sci..

[35] Droogsma E., van Asselt D., Bieze H., Veeger N., De Deyn P.P. (2015). The relationship of weight change trajectory with medial temporal lobe atrophy in patients with mild Alzheimer’s disease: Results from a cohort study. Alzheimers Res. Ther..

[36] Stenholm S., Head J., Aalto V., Kivimaki M., Kawachi I., Zins M., Goldberg M., Platts L.G., Zaninotto P., Magnusson Hanson L.L. (2017). Body mass index as a predictor of healthy and disease-free life expectancy between ages 50 and 75: A multicohort study. Int. J. Obes..

[37] Berrington de Gonzalez A., Hartge P., Cerhan J.R., Flint A.J., Hannan L., MacInnis R.J., Moore S.C., Tobias G.S., Anton-Culver H., Freeman L.B. (2010). Body-mass index and mortality among 1.46 million white adults. N. Engl. J. Med..

